# Exercise effects on neuropsychiatric symptoms and quality of life in mild cognitive impairment: a systematic review and meta-analysis

**DOI:** 10.3389/fneur.2024.1447734

**Published:** 2024-10-14

**Authors:** Liang Chen, Sung Min Kim

**Affiliations:** ^1^Department of Physical Education, Human-Tech Convergence Program, Hanyang University, Seoul, Republic of Korea; ^2^Department of Sport Science, Hanyang University, Seoul, Republic of Korea; ^3^Center for Artificial Intelligence Muscle, Hanyang University, Seoul, Republic of Korea

**Keywords:** exercise, mild cognitive impairment, neuropsychiatric symptoms, life quality, mind–body exercise

## Abstract

**Background:**

Exercise is considered as a cost-efficient option for individuals with mild cognitive impairment (MCI). Although the potential benefits of exercise for improving cognitive function are recognized, its impact on neuropsychiatric symptoms (NPS) and the quality of life (QoL) in individuals with MCI remains unclear.

**Objective:**

This study aims to investigate the effects of exercise interventions on NPS, including depression, anxiety, sleep disorders, and the quality of life in individuals with MCI.

**Results:**

There were 17 randomized controlled trials (RCTs) involving 1,575 participants were included. The findings indicate a small but significant positive effect of exercise on depression (standardized mean difference [SMD] = −0.47, 95% confidence interval [CI]: −0.73 to −0.21), but the quality of the evidence is low. Mind–body exercises were relatively more effective in alleviating depression. No significant improvements were observed in sleep disorders (SMD = −1.27, 95% CI: −2.80 to 0.26). Exercise improved anxiety in one study but had no effect in the other. It also improved quality of life in seven studies, with two showing no effect.

**Conclusion:**

The findings suggest that exercise, particularly mind–body exercises, can improve depression in individuals with MCI. However, the effect of exercise on anxiety, sleep disorders, and quality of life is still uncertain, necessitating additional research. These findings underscore the significance of integrating customized exercise programs in managing MCI to boost quality of life and mitigate NPS.

**Systematic Review Registration:**

https://www.crd.york.ac.uk/prospero/. ID: CRD42023445369.

## 1 Introduction

Mild cognitive impairment (MCI) is an intermediate stage between normal aging and dementia onset, and it is classified into two subtypes: amnestic MCI (aMCI) and non-amnestic MCI (naMCI) (1, 2). The clinical manifestations include memory loss, language ability damage, attention difficulties, and executive function impairment. Moreover, a considerable number of individuals with MCI also exhibit neuropsychiatric symptoms (NPS) (3). Research shows that approximately 35–85% of individuals with MCI have at least one NPS, with depression exhibiting the highest reported NPS prevalence rate (83% for aMCI and 73% for naMCI) (4). Anxiety and sleep disorders are also common in MCI, with prevalence rates exceeding 70 and 39%, respectively (5). In addition, negative psychological states significantly impact the quality of life in individuals with MCI (6–8). Inadequate or delayed treatment has an adverse impact on cognitive functioning and the general state of health (9–11).

In recent years, treatments for individuals with MCI have generally been categorized into pharmaceutical and non-pharmaceutical therapies. Existing evidence suggests that pharmacotherapy (represented by cholinesterase inhibitors) shows some efficacy in preventing and treating cognitive decline in MCI, but there is still a lack of high-quality clinical evidence (12–14). Fortunately, some meta-analyses have demonstrated that non-pharmaceutical interventions, especially exercise, can enhance global cognitive function, memory and executive function (15–17). Additionally, studies have found that exercise combined with cognitive training can improve motor performance in MCI patients, including balance, gait, and mobility (18, 19).

However, whether it is pharmacotherapy or non-pharmacotherapy, more focus has been placed on treating cognitive domain impairment than on addressing NPS in individuals with MCI. As a safe and economical intervention, exercise has played an important role in improving mood states, such as depression, anxiety, tension, and confusion (20–22). Therefore, exercise intervention could be a new, easy, and convenient therapy for improving NPS with MCI. Research has shown that exercise interventions have notable impacts on reducing anxiety (23) and alleviating depression (24) in MCI. Moreover, a meta-analysis of six studies demonstrated that exercise was an effective intervention for alleviating depression in older adults with MCI (25). Similar results were also supported by another meta-analysis (26).

Although the above research has highlighted the beneficial effects of exercise on specific NPS, controversies still remain. Liu et al. (27) indicated that the impact of dance intervention on depression, anxiety, and quality of life in persons with MCI requires further verification (27). Another study has indicated that traditional Chinese exercise has no effect on depression (28). Therefore, previous meta-analyses on this area had notable limitations. First, they included a limited number of studies, exhibited high heterogeneity, and suffered from methodological quality issues, undermining the persuasiveness of the findings (25, 29, 30). Second, the majority of the related meta-analyses have focused on the cognitive domain, rather than the NPS. Third, despite the fact that several reviews have reported the effect of exercise interventions on NPS with MCI, all of them solely focused on partial psychological symptoms, particularly depression. Finally, the varying effectiveness of different interventions and conditions necessitates subgroup analyses for a meaningful evaluation of intervention outcomes.

Therefore, considering both the limitations of prior research and the substantial advantages of exercise on quality of life, this study aims to evaluate the effects of exercise on common NPS and overall quality of life in individuals with MCI.

## 2 Materials and methods

### 2.1 Protocol and registration

This meta-analysis was reported according to the Preferred Reporting Items for Systematic reviews and Meta-Analyses (PRISMA) instructions (31). It has been recorded in the International Prospective Register of Systematic Reviews (PROSPERO) with the registration ID CRD42023445369.

### 2.2 Information sources and search

The search process involved two steps. First, an independent literature search was conducted within the database, verifying each step of the process. The trials available in the Randomized Controlled Trials Registry platform but not yet published were also sought. The development of literature supplements and searches utilized the references from the articles that had been screened. Subsequently, full portions of research that potentially met the inclusion criteria were obtained for a comprehensive eligibility assessment. Second, the retrieved data was imported into Endnote X9 software to eliminate any duplicates. The research was screened based on predetermined inclusion and exclusion criteria. We conducted a search in the following databases: Web of Science, Scopus, PubMed, APA PsycINFO, Embase, Cochrane Library, and MEDLINE (from inception to 18 February 2024). To ensure a comprehensive search and minimize the risk of overlooking relevant studies, we developed a search strategy that incorporated a variety of pertinent major search terms (Medical Subject Headings [MeSH] terms) and free-text keywords. Trials on depression, sleep disorders, anxiety, and quality of life effects were screened during a subsequent literature search. Supplementary Table 1 outlines the screening methodology.

### 2.3 Eligibility criteria and study selection

Inclusion criteria followed are (1) Participants: screened individuals aged 50 years or older with a primary diagnosis of MCI, as defined by the Research Diagnostic Criteria, or adults with MCI identified through validated screening measures, scoring below normal threshold values (e.g., Montreal Cognitive Assessment [MoCA]). (2) Interventions: investigated the use of exercise in treating MCI, defining it as a deliberate, organized, repeated, and purposeful physical activity designed to enhance or sustain physical fitness (32). (3) Comparisons: included no treatment, usual care, placebo, waitlist, or health education. (4) Follow-up: for at least 8 weeks or more (from the trial baseline assessment). (5) Outcomes: at least one examination result related to depression, anxiety, sleep disorder, or quality of life was reported. (6) Design: Randomized Controlled Trials (RCTs).

Excluded criteria followed are (1) the absence of MCI participants; (2) the absence of non-exercise intervention; (3) the absence of RCTs; (4) the absence of full-text available; (5) the inability to extract outcome data; and (6) the absence of studies (such as conference abstracts, animal experiments, etc.).

### 2.4 Data extraction

Data was extracted using a pre-prepared form by one collaborator (LC), with the accuracy subsequently verified by another collaborator (SMK). The information obtained from the qualifying research comprised the following: study characteristics, participant characteristics, interventions, and outcome measurement information. In cases where incomplete statistics were provided, the mean and standard deviation were estimated using the sample size, median, quartile, and other available reported data (33–35).

Furthermore, exercises were grouped based on the objectives of this study and previous reviews. There were four types: (1) Aerobic exercise (AE; enhancing cardio-respiratory fitness and including activities, such as walking, swimming, and cycling) (17, 36, 37). (2) Mind–Body exercise (MBE; enhancing mind–body coordination and awareness, such as yoga, dance, and Tai chi) (17, 36–38). (3) Multicomponent exercise (ME; a minimum of two exercise modalities, such as walking and limb balance training, running and skipping) (36, 37). (4) Physical and Cognitive Training (PCT; combines exercise and cognitive training, such as walking and memory training or sport stacking) (36, 39). Subsequently, subdivisions within different exercise categories were organized based on intervention frequency, duration, and session. The consistency of the extracted data was evaluated by the Intra-class Correlation Coefficient method before addressing the difference, resulting in an outcome of 0.97, which signifies high consistency in data extraction (40).

### 2.5 Risk and bias

The risk of bias in the qualifying trials was assessed using Cochrane’s Risk of Bias Tool 2.0 (RoB 2) (41, 42). Two collaborators independently assessed six aspects, including the randomization process (D1), deviations from the intended interventions (D2), missing outcome data (D3), measurement of the outcome (D4), selection of the reported result (D5), and overall bias (D6). The risk of bias was categorized as “low risk,” “some concern,” or “high risk” based on their assessments for each domain. Discrepancies between the researchers’ assessments were discussed with a third party. We used Cohen’s kappa coefficient to assess interrater reliability, which indicated a high level of agreement with an overall kappa value of 0.91. Specifically, the kappa values were 0.88 for D1, 0.73 for D2, 0.86 for D6, 1.00 for D3, D4, and D5 (43). Studies were not excluded based on the high risk of bias.

### 2.6 Statistical analyses

The meta-analysis was carried out using Review Manager 5.3 and Stata 17.0 software. Effect sizes were reported as mean differences with 95% confidence intervals (CIs). When outcomes were measured using varying scales with similar constructs, the standard mean difference (SMD) was used (44). Four categories of heterogeneity were determined based on the *I*^2^ index: low (*I*^2^ ≤ 25%), moderate (25–50%), high (50–75%), and extremely high (*I*^2^ > 75%) (45). A random-effect model was employed when *I*^2^ was >50%; otherwise, a fixed-effect model was used. Furthermore, due to high heterogeneity, subgroups were analyzed, based on factors such as exercise frequency, session, and duration. Publication bias was evaluated by Egger’s regression asymmetry test (46). Funnel plots were generated and visually inspected for outcomes with 10 or more included studies to detect potential asymmetry in the distribution of studies.

### 2.7 Quality of evidence

We used the GRADE (Grading of Recommendations, Assessment, Development, and Evaluation) framework to evaluate the evidence quality (47), which covered five key areas, such as study limitations, inconsistency, imprecision, indirectness, and publication bias. Two researchers, LC and SMK, independently conducted the evaluations using GRADEpro Guideline Development Tool software,^1^ categorizing evidence quality into four levels: very low, low, moderate, and high. In the GRADE approach, the initial quality level of included studies is considered high. If one risk factor is present and severe, the evidence quality is reduced by one level; if the risk is very severe, the evidence quality is reduced by two levels. As this analysis included only RCTs, conditions that could potentially enhance the quality level, such as large sample sizes, high effect sizes, and the presence of confounding factors, were not applicable. Each outcome’s evidence quality was evaluated using GRADE standards (48), and all decisions to downgrade evidence quality were justified with annotations. The two researchers reached full agreement in their assessments.

## 3 Results

### 3.1 Study selection

**Figure 1:**
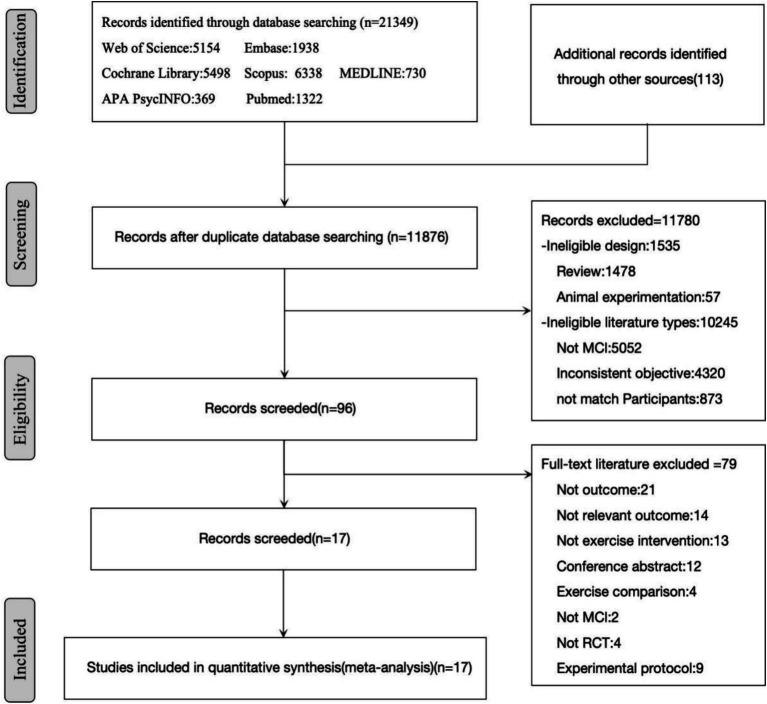
Study selection process flowchart.

From databases, reference lists, and other sources, 21,462 studies were retrieved. Among these, 9,586 duplicate records were removed, leaving 11,876 studies for further examination. After reviewing titles and abstracts, 11,780 records were excluded as they did not meet the eligibility criteria. After examining the full text of 96 articles, 79 records were eliminated. Finally, 17 studies were included (Figure 1).

### 3.2 Study characteristics

A total of 17 studies, encompassing 1,575 participants, are briefly summarized in Table 1. The studies, conducted between 2007 and 2022, had participants with an average age of 71.83 years. Asia had the most articles with 10, followed by Europe with 4 and the Americas with 3.

**Table 1:** Study characteristics.

No	References	Country (region)	Setting	Sample size (I:C)	Gender type (F/M) (I:C)	Mean age (I:C)	Intervention	Duration (weeks)	Frequency (times)	Session (min)	Comparison	Outcomes
1	(51)	Dutch	Community	71:67	48/29:55/19	75 /75	AE (Walking)	48	2	60 min	placebo pills	D-QoL
2	(59)	Hong Kong	Social centers	147:131	34/113:29/102	75.5/75.4	ME (Tai Chi + static bicycle riding)	48	3	60 min	social activities	CSDD
3	(59)	Hong Kong	Social centers	132:131	28/104:29/102	76.3/75.4	PCT (Yoga + Writing)	48	3	60 min	social activities	CSDD
4	(63)	Slovak	Community	40:40	22/18:19/21	68/65.9	PCT (Balance exercise + CogniPlus training program)	10	7/2	30 min	usual care	QL-Index
5	(53)	US	Longevity Center	38:41	25/13:27/14	68.1/67.6	MBE (Yoga)	12	1	60 min	Memory training	GDS-30
6	(54)	Greek	Community	66:63	\	65.9/ 67.9	MBE (International Ballroom Dancing)	40	2	60 min	waiting list	GDS-15
7	(57)	China	Hospital	27:31	14/15:10/31	70.3 /69.0	MBE (Specially designed dance)	12	3	35 min	usual care	GDS-15 SF-36
8	(58)	Turkey	Nursing home	30:30	18/12:17/13	72.2/70.7	ME (Rhythmic exercise + Walking)	20	4/3/7	80 min	social activities	PSQI
9	(79)	China	Community	60:60	48/12:42/18	76.2/75.3	AE (Aerobic stepping exercise)	16	3	60 min	health education	GDS-30 QOL-AD-C PSQI
10	(49)	Brazilian	Primary Healthcare Units	26:26	6/20:6/20	72.6 /71.9	AE (Walking^a^)	24	2	60 min	usual care	GDS-15
11	(56)	China	Community Healthcare Center	57:54	21/36:22/32	68.4/68.2	ME (Structured limbs-exercise)	24	3	60 min	health education	GDS-15 PSQI
12	(56)	China	Nursing home	33:33	7/26:12/21	81.1/81.1	MBE (Chinese square dancing)	12	3	40 min	usual care	GDS-15 SF-12
13	(61)	Hong Kong	Community	5:6	2/4:1/5	70.7/74.5	PCT (Tai Chi + Rummikub)	12	3	90 min	health education	GDS-15 GAS-20 EQ-5D
14	(60)	Korea	Community	13:13	4/8:4/8	70.2/71.8	PCT (Aerobic exercise^b^ + Cognitive activities^c^)	12	2	90 min	health education	SGDS-K
15	(52)	China	Nursing Home	62:47	\	76.6/75.9	MBE (Chinese square dancing)	18	3	30 min	usual care	GDS-15 SF-12
16	(55)	US	Hospital and Community	25:21	10/15: 18/3	71.6/71.7	MBE (Yoga)	12	2	60 min	health education	CES-D SF-36 STAI-T
17	(65)	China	Hospital	9:11	5/4:4/7	72.0/70.5	PCT (Sport Stacking)	12	5	30 min	usual care	GDS-30 PSQI
18	(64)	Philippines	Community	30:30	6/24:8/22	63.3/64.3	PCT (Zumba dance + cognitive training^d^)	12	3	60 min	health education	GDS-SF PWB

This table was created by collecting data from patients with mild cognitive impairment, which refers to a decline in cognitive function that does not yet meet the criteria for dementia. I, Intervention group; C, Control group; CSDD, Cornell Scale for Depression in Dementia; CED-S, Centers for Epidemiological Studies Depression Scale; D-QoL, Dementia Quality of Life questionnaire; EQ-5D, EuroQoL 5-D Questionnaire; GDS-30, Geriatric Depression Screening Scale; GDS-15, Geriatric Depression Scale with a maximum score of 15; GDS-SF, Geriatric Depression Scale-Short Form; GAS-20, Geriatric Anxiety Scale with a maximum score of 20; PSQL, Pittsburgh Sleep Quality Index; PWB, 14-item Perceived Well-being; QOL-AD, Quality of Life in Alzheimer’s disease; QOL-AD-C, Quality of Life in Alzheimer’s disease Chinese version; QL-Index, Quality of Life Index; STAI-T, State–Trait Anxiety Inventory-Trait; SF-12, Short-Form 12 health survey; SF-36, 36-item Short Form Health Survey; SGDS-K, Korean version of the geriatric depression scale; AE, Aerobic exercise, aimed at enhancing cardiorespiratory fitness includes activities such as walking, running, swimming, or cycling; MBE, Mind–Body exercise, aimed at enhancing mind–body coordination and awareness, includes yoga, dance, or Tai chi; ME, Multicomponent exercise, involving the integration of a minimum of two exercise modalities, such as walking and limb balance training, running, and skipping; PCT, Physical and cognitive exercise, a combination of exercise and cognitive training, such as walking and memory training or sport stacking. F, Female; M, Male.

a Primarily focus on aerobic walking, with added lower body training.

b Walk in line, toe rock-paper-scissors, step box, thera-band exercise, body rock-paper-scissors, ladder, etc.

c Speaking, counting, word games, performing fast, uncomplicated numerical calculations, and playing a simple memory span game.

d Arm-clock exercises, recall, and spelling.

Aerobic exercise interventions (e.g., walking and stepping) were reported in 3 articles (49–51), mind–body exercise interventions (e.g., Kundalini yoga and Chinese square dance) in 6 articles (52–57), multicomponent exercise interventions (e.g., rhythmic exercise combined with walking, Tai Chi paired with static bicycle riding, and limb exercises) in 3 articles (56, 58, 59), and physical and cognitive exercise interventions (e.g., Rummikub combined with Tai Chi, cognitive tasks paired with Zumba dance, and cognitive activities combined with aerobic exercise) in 6 articles (59–65). Lam et al. (59) identified two types of exercise interventions, thereby categorizing them into distinct exercise types: multicomponent exercise and cognitive and physical training. In addition, the trials had an exercise intervention activity duration ranging between 10 and 48 weeks. The intervention lasted for 12 weeks or less in 9 studies, 12–24 weeks in 5 studies, and more than 24 weeks included 3 studies. Additionally, follow-up periods after intervention were reported in only three studies (53, 57, 64).

The effectiveness of interventions for depressive symptoms was reported in 14 articles, using all kinds of assessment tools. The Geriatric Depression Scale (GDS), which has a maximum score of 15 (GDS-15), the most frequently used tool, was used in seven studies (49, 52, 54, 56, 57, 61). Following this, the Geriatric Depression Screening Scale (GDS-30) was used in three studies (50, 53, 65), the Cornell Scale for Depression in Dementia (CSDD) in one study (59), and the Korean version of the Geriatric Depression Scale (SGDS-K) in one study (60), the Centers for Epidemiological Studies Depression (CES-D) Scale in one study (55), and the Geriatric Depression Scale-Short Form (GDS-SF) in one study (64). These tools not only share a similar construct and high structural similarity (66, 67) but also have some differences. GDS-15 and GDS-SF are short and suitable for quick screening, while GDS-30 is more detailed and is often used for comprehensive assessment. The Korean version of the GDS, SGDS-K, is culturally adapted and validated. The CSDD is specifically designed for dementia patients, including cognitive impairment. The CES-D is used for a broader population, especially in epidemiological studies, to assess depressive symptoms. In clinical practice, different measurement tools can be selected based on specific needs. Although different tools to measure depression, these tools share a similar construct. Therefore, we used the SMD method for analysis.

Furthermore, in 9 articles, researchers assessed the quality of life using various tools, including EuroQoL 5-D (EQ-5D) (61), the Quality of Life Index (QL-Index) (63), the Short Form Health Survey-36 (SF-36) (57), the Quality of Life-Alzheimer’s disease (QoL-AD-C; Chinese version) (50), the Dementia Quality of Life questionnaire (D-QoL) (51), the 14-item Perceived Well-being (PWB) (64), the 36-item Short Form Health Survey (SF-36; Chinese version) (55), and the Short-Form 12 health survey (SF-12) (52, 56). Although these tools are designed to assess quality of life, they differ in their internal structure, specific target populations, and assessment content. For example, the EQ-5D is suitable for a broad population, QoL-AD-C and D-QoL focus on dementia and Alzheimer’s patients; the QL-Index provides a comprehensive evaluation of health and social aspects; SF-36 and SF-12 assess eight health dimensions; and the PWB assesses individual well-being.

Finally, sleep quality was assessed in 4 articles using the Pittsburgh Sleep Quality Index (PSQI) (50, 56, 58, 65), which covers multiple dimensions such as sleep duration, efficiency, and disturbances, making it suitable for a broad population. Anxiety symptoms were also the focus of the two articles. They were assessed using the Geriatric Anxiety Scale (GAS-20) (61), which is specifically designed for the elderly, and the State–Trait Anxiety Inventory-Trait (STAI-T) (55), which measures both situational and persistent anxiety. These tools are similar in their assessment of specific mental health aspects but differ in their assessment content and internal structure.

### 3.3 Risk of bias within studies

The risk of bias for each included RCT was evaluated using RoB 2. Although Cohen’s kappa coefficient indicated a high level of agreement among the researchers (0.91), discrepancies in the risk of bias assessment necessitated a final decision through consultation with a third party (SWY). Consequently, three studies were identified as having a low risk (50, 59, 65), six studies were identified as having a high risk (49, 51, 52, 54–56), and eight studies were identified as having some concerns (53, 56–58, 60–64).

**Figure 2:**
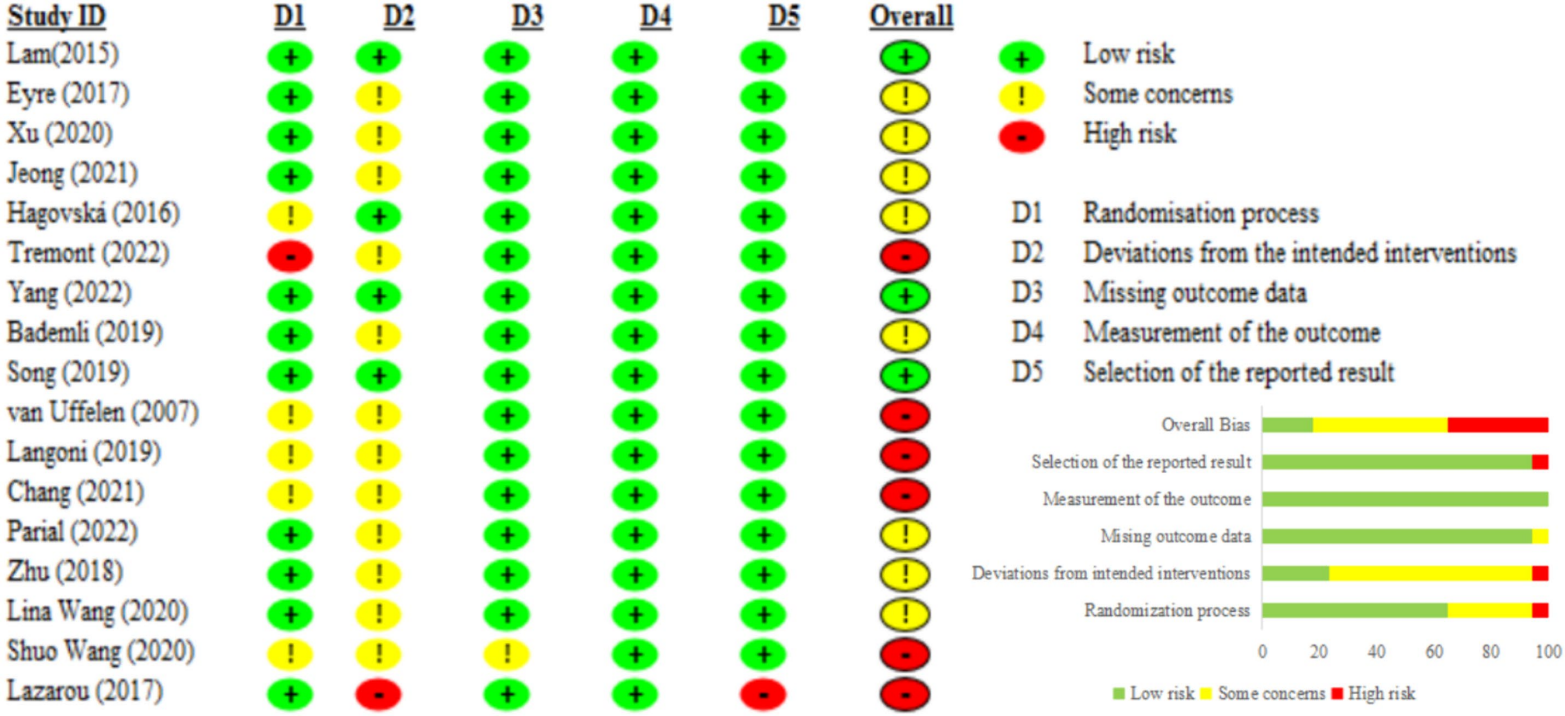
Risk of bias assessment.

The studies classified as high risk presented significant issues in two aspects: (1) at least one domain had high risk, such as the lack of allocation concealment (55) and the absence of an appropriate analytical method to address attrition during the intervention (54) (2) two domains had some concerns, including inadequate details on allocation concealment and deviations from intended interventions (49, 51, 52) and high attrition rates (56). The studies with some concerns were noted to lack detailed information on allocation concealment (63) and deviations from the intended interventions (53, 56–58, 60, 61, 64) (Figure 2).

#### 3.3.1 Regarding the risk of bias in randomization

All studies utilized randomization methods to assign participants, but the specific methods varied. Some studies used computer-generated random codes (49–51, 53, 54, 56–59, 61–65), while others used methods such as drawing lots (52) or coin tossing (56), and urn randomization (55). However, some studies did not specify the randomization methods used in these studies (60). Additionally, only six studies provided detailed descriptions of the allocation concealment procedures (50, 53, 54, 56, 57, 61).

#### 3.3.2 Regarding deviation from intended interventions

Five studies adopted the intention-to-treat (ITT) analysis method (50, 51, 56, 59, 64), while other studies did not provide detailed information on how they handled deviations from the intended interventions.

### 3.4 Synthesis of results

When conducting data screening and processing, it was found that only a few studies reported the post-intervention effects, which limited our ability to better analyze the long-term effects. Consequently, we opted to assess the immediate impact of the intervention on individuals with MCI at the conclusion of the intervention period.

#### 3.4.1 Depression

**Figure 3:**
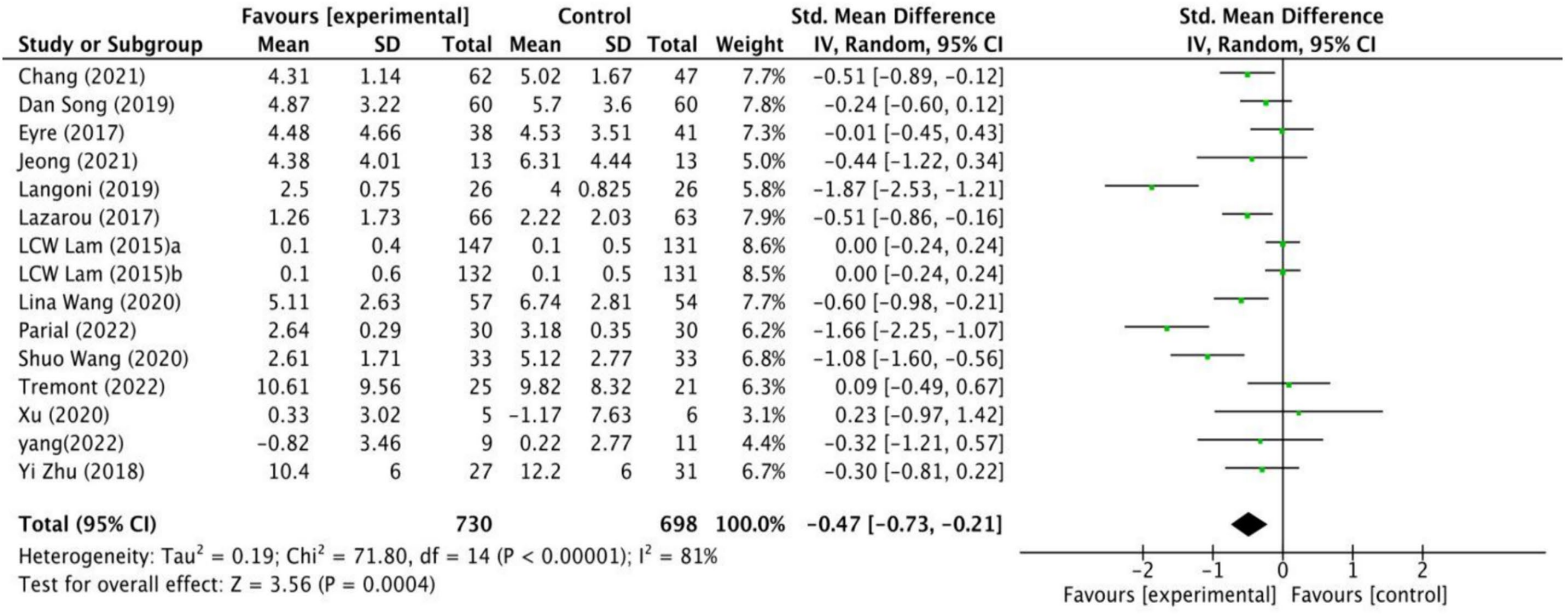
The effect of exercise on depression.

The impact of exercise interventions on depression among MCI patients has been documented in 14 studies (Figure 3). The random effects model was employed due to the substantial heterogeneity (*I*^2^ = 81%, *p* < 0.00001). The results show a statistically significant disparity between the exercise intervention and the control group in terms of depression symptoms among MCI patients, with a mean effect size of (SMD = −0.47, 95% CI: −0.73 to −0.21, *Z* = 3.56, *p* = 0.0004). Additionally, potential sources of high heterogeneity were explored through subgroup analyses.

##### 3.4.1.1 Subgroup analyses

**Figure 4:**
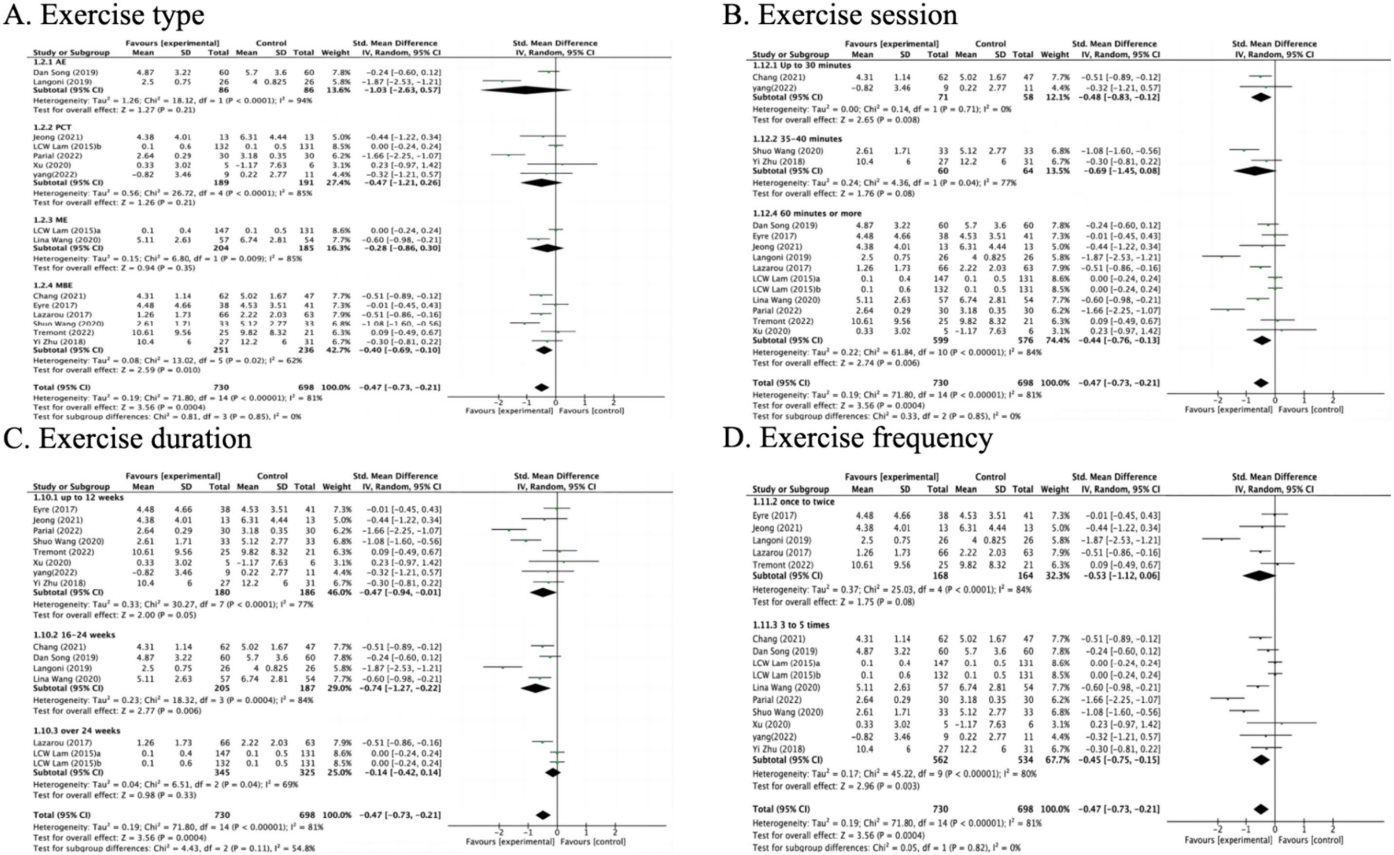
The effect of exercise type on depression (subgroup analyses).

In the subgroup analysis of exercise types, MBE(6), PCT(5), ME(2) and AE(2) types were included (Figure 4A). Specifically, it was found that the MBE intervention type had a significant impact on improving depression compared to the control group (SMD = −0.40 95% CI: −0.69 to −0.10, *Z* = 2.59, *p* = 0.010). However, no statistically significant differences were observed in the intervention types of AE (SMD = −1.03, 95% CI: −2.63 to 0.57, *Z* = 1.27, *p* = 0.21), PCT (SMD = −0.47, 95% CI: −1.21 to 0.26, *Z* = 1.26, *p* = 0.21), or ME (SMD = −0.28, 95% CI: −0.86 to 0.30, *Z* = 0.94, *p* = 0.35).

Exercising for 30 min or less per week (SMD = −0.48, 95% CI: −0.83 to −0.12, *Z* = 2.65, *p* = 0.008) and 60 min or more per week (SMD = −0.44, 95% CI: −0.76 to −0.13, *Z* = 2.74, *p* = 0.006) was associated with improvements in depression (Figure 4B). Additionally, exercise programs lasting 12 weeks or less (SMD = −0.47, 95% CI: −0.94 to −0.01, *Z* = 2.00, *p* = 0.05) and those between 16 and 24 weeks (SMD = −0.74, 95% CI: −1.27 to −0.22, *Z* = 2.77, *p* = 0.006) were found to be effective (Figure 4C). Furthermore, exercising 3 to 5 times per week (SMD = −0.45, 95% CI: −0.75 to −0.15, *Z* = 2.96, *p* = 0.003) also correlated with reductions in depression (Figure 4D).

##### 3.4.1.2 Publication bias

**Figure 5:**
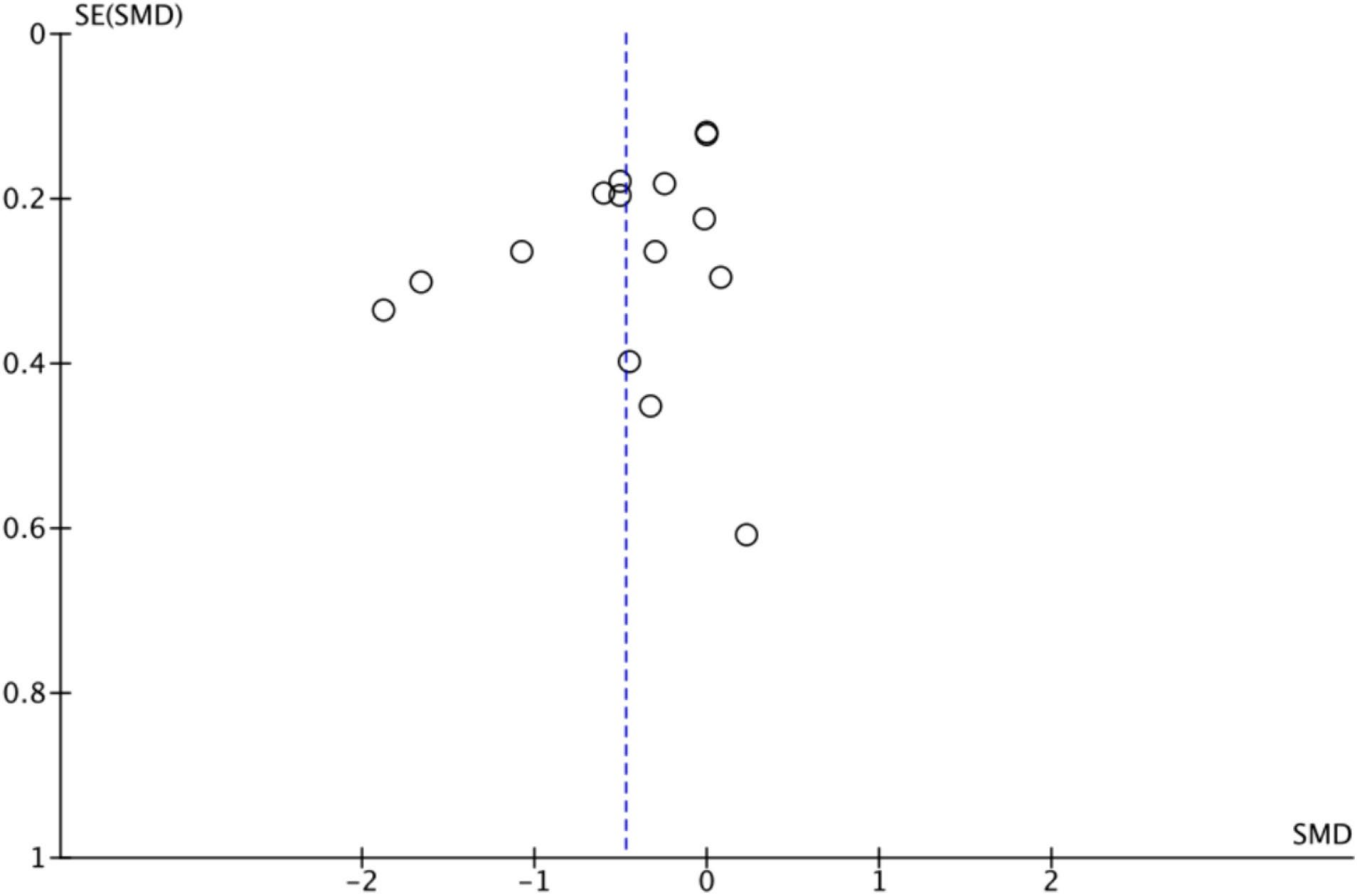
Funnel plot for the exercise therapy on depression.

Figure 5 shows that the effect size of exercise therapy on depression is symmetrical. In addition, to enhance the objectivity of the results, Egger’s regression model was applied to assess publication bias. The results demonstrated no statistically significant difference and indicated no publication bias (*t* = −1.90, df = 20.21, *p* = 0.08).

#### 3.4.2 Sleep quality

**Figure 6:**
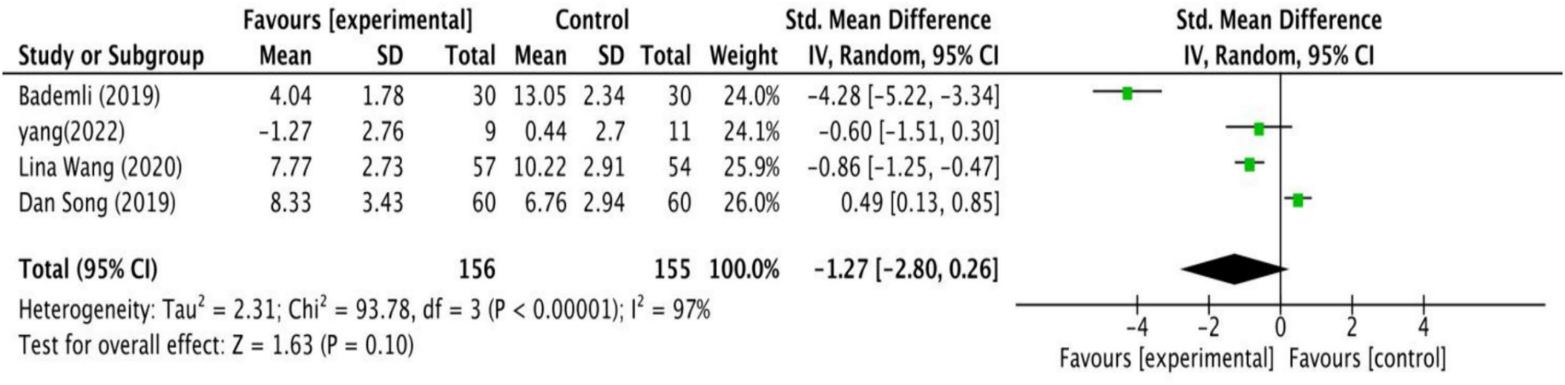
The effect of exercise on sleep quality.

Four studies examined how exercise interventions affected the quality of sleep in MCI patients. As shown in Figure 6, the analysis revealed no significant differences between intervention and control groups (SMD = −1.27, 95% CI: −2.80 to 0.26, *Z* = 1.63, *p* = 0.10).

#### 3.4.3 Anxiety

Two studies investigated the effects of exercise interventions on anxiety in individuals with MCI. A small-sample study found preliminary improvements in anxiety (61), while the other study showed no significant effect (55). The interventions consisted of two types, PCT and MBE.

#### 3.4.4 Quality of life

A total of 9 RCT studies focused on exercise interventions in patients with MCI: 2 on AE, 3 on PCT, and 4 on MBE. Seven studies showed that exercise interventions significantly improved quality of life (50–52, 56, 57, 63, 64), while two studies showed no improvement (55, 61).

### 3.5 Quality of evidence within studies

Based on the GRADE assessment, the quality of the evidence for the effects of exercise interventions on depression and sleep quality in MCI patients is low and very low (Table 2). In the analysis of depression, although results were combined from 14 studies, the overall quality of evidence remains low due to high heterogeneity (*I*^2^ = 81%) and the presence of several high-risk bias studies. For sleep quality, 50% of the studies raised concerns about bias, and the heterogeneity was 97%. The total sample size was below 400, resulting in wide confidence intervals. These factors contribute to a low quality of evidence.

**Table 2:** Research quality grade assessment results.

Certainty assessment							No of patients		Effect		Certainty	Importance
No of studies	Study design	Risk of bias	Inconsistency	Indirectness	Imprecision	Other considerations	Exercise	Comparison	Relative (95% CI)	Absolute (95% CI)		
Depression_MCI (assessed with SMD)												
14	randomised trials	serious^a^	serious^b^	not serious	not serious	none	730	698	–	SMD – 0.47 SD lower (−0.73 lower to −0.21 lower)	⨁⨁◯◯ Low	Important
Sleep quality_MCI (assessed with PSQI)												
4	randomised trials	serious^c^	serious^d^	serious^e^	not serious	none	156	155	–	SMD – 1.27 SD lower (−2.8 lower to 0.26 higher)	⨁◯◯◯ Very low	Important

CI, confidence interval; SMD, standardized mean difference.

a Some studies have the following limitations: deviations from the intended interventions, absence of an appropriate analytical method to address attrition during the intervention, and lack of allocation concealment.

b *I2 > 75%; high heterogeneity indicates substantial inconsistency among the study results.*

c Deviations from the intended interventions (56, 58).

d *I2 > 75%; high heterogeneity and inconsistencies in effect size.*

e The total population size is less than 400.

## 4 Discussion

This study examined the effects of exercise on depression, quality of life, anxiety, and sleep in individuals with MCI. The meta-analysis results indicated that the exercise group had superior outcomes for depression than the non-exercise group. However, exercise had no significant impact on sleep. Additionally, while several RCTs have demonstrated that exercise can enhance anxiety or quality of life, others are not entirely consistent.

Our study results indicate that exercise can help alleviate depression in patients with MCI, consistent with previous meta-analyses (25, 30, 61). Furthermore, we analyzed the effects of various exercises (AE, ME, PCT, and MBE) on depressive symptoms. AE, such as walking and stepping, has been shown to reduce depressive symptoms (49, 50). ME, which integrates activities such as Tai Chi and static cycling, has been demonstrated to enhance both physical and mental health, thereby alleviating depressive symptoms (59). PCT, which integrates physical activity with cognitive tasks, has shown promise in simultaneously engaging the body and mind, thereby reducing depressive symptoms and enhancing overall cognitive function and emotional well-being (64).

However, our meta-analysis results show that mind–body exercises outperform other types of exercises in alleviating depressive symptoms, and demonstrate a small but significant effect (68). This finding is similar to that of a previous meta-analysis (25). Mind–body exercises, which are characterized by full-body stretching and relaxation, focus attention, and rhythmic breathing (38), integrate both mental and physical training. Compared to other types of exercise, mind–body exercises place a greater emphasis on participants’ internal experiences and proprioception, achieving similar intervention effects to high-intensity exercise in improving depressive symptoms (69).

In the RCTs included in this study, the frequency of exercise interventions varied from one to five times per week, with the majority of reports indicating a frequency of three times per week. Exercise frequency of three or more times per week has alleviated depression in individuals with MCI. Perraton et al. (70) also found that exercising three times per week significantly improved depressive symptoms (70). This frequency enhances physical fitness and enables the body in adapting to the stress induced by exercise while also providing adequate recovery time. The exercise duration in the RCTs varied from 12 to 48 weeks, with 12-week interventions being the most commonly reported. Interventions that lasted less than 24 weeks showed improvements in depressive symptoms, whereas those that lasted longer than 24 weeks did not achieve the expected results. The ineffectiveness of longer interventions may be attributed to decreased participant motivation and increased psychological and physical fatigue, which may impair the effectiveness of the intervention. In contrast, shorter interventions, such as those lasting 12 weeks, are easier to control for experimental variables and maintain high quality. As a result, they are more commonly selected and reported. Additionally, studies have shown that each additional 10 min of exercise enhances the antidepressant effect, with approximately 60 min being optimal (71). Our research also suggests that exercise interventions of 30 min and 60 min or more are effective, with the highest number of reports for 60-min interventions. Therefore, when designing exercise intervention programs, it may be preferable to choose a regimen lasting 12 weeks with sessions spreading three times per week and each session lasting 60 min.

However, it is worth noting that the overall quality of evidence is considered to be low. There are several limitations present in some studies, such as deviations from the intended interventions, a lack of appropriate analytical methods to address attrition during the intervention, and inadequate allocation concealment. Furthermore, some studies did not fully adhere to standards for allocation concealment and ITT analysis, which may have led to an overestimation of the exercise effects. Thus, it is important to interpret the results with caution.

Anxiety, another common neurological symptom in MCI patients, has been less extensively studied than depression in MCI patients. Although some research has indicated that exercise can have beneficial effects on mood and anxiety (72, 73), the impact of exercise on subsequently affecting anxiety in MCI patients remains unclear. This study refrained from conducting a meta-analysis of the impact of exercise interventions on anxiety symptoms in patients with MCI, as it included only two studies and faced inconsistencies in measurement methods. One study with some concerns about bias, which utilized a PCT regimen combining Tai Chi with Rummikub, showed a positive intervention effect (61). The intervention was conducted thrice a week, with each session lasting 90 min over a 12-week period. However, the small sample size of only 11 participants may hinder the interpretability of the intervention’s effectiveness. Another study, identified as having a high risk of bias, involved MBE in the form of yoga and reported no significant effect (55). This regimen was carried out twice weekly, with each session lasting 60 min, also over 12 weeks. Despite the inconsistent intervention outcomes, it is interesting that both studies chose MBE as their intervention approach (Tai Chi, Yoga). This preference may be due to the potentially favorable effects of MBE on anxiety (74, 75). Nonetheless, due to the small sample size, more rigorous and extensive RCTs are necessary to better explore the impact of exercise on anxiety in patients with MCI.

For sleep disorders, a meta-analysis reported that exercise can improve PSQI scores in adults (76), but the impact of exercise on sleep quality in patients with MCI remains uncertain due to inconsistent findings across studies (65). Our study synthesized the findings of other four studies and concluded that exercise did not significantly improve sleep quality in MCI patients. The studies involved primarily limited exercise types, including one on AE (Aerobic stepping exercise), one on PCT (Sport Stacking), and two on ME (Rhythmic exercise and walking, Structured limbs-exercise), with most exercises being simple and repetitive. Notably, there was a lack of research on the effects of MBE. The intervention protocols varied greatly, with durations ranging from 15 to 24 weeks, frequency from 3 to 7 times per week, and session length from 30 to 80 min. Such variability in the protocols and differences in participant characteristics (e.g., age, country), may have contributed to the high heterogeneity observed in the meta-analysis. This heterogeneity, combined with the small sample sizes of the included studies, led to the overall quality of evidence being considered to be very low (48). Therefore, future exercise intervention studies need to improve research design and enhance study quality in order to increase the credibility of the research conclusions.

Compared to individuals without MCI, the quality of life in individuals with MCI significantly declines (77). This decline is related to neuropsychiatric symptoms, functional deterioration, and cognitive decline (78). Due to inconsistencies in measurement methods and disparities in internal structure, we were unable to conduct a meta-analysis on the impact of exercise interventions on quality of life. However, our investigation has revealed that the majority of studies indicate a positive effect of exercise on quality of life. The most common exercise regimen was 12 weeks in duration, with sessions spread three times per week, each session lasting 60 min. Dance-based interventions were the most prevalent, appearing in four studies, with two of them focusing on Chinese square dancing. Furthermore, studies have shown that depression is significantly related to other factors (79). Research by Kang and Lee (80) supports this view. They analyzed the factors affecting the quality of life of 348 individuals with MCI in South Korea and found that depression is a strongly associated factor. Our meta-analysis also revealed that mind–body exercises, particularly square dancing, are more effective in alleviating depression. It is possible that socially engaging exercises like square dancing improve depression in individuals with MCI, indirectly enhancing their quality of life (56). Therefore, exercise might act as an intermediary factor between MCI symptoms and the enhancement of quality of life, with dance-based interventions potentially offering more significant benefits (52). However, four of the included studies were classified as high-risk, while the others had some concerns. Future research on interventions for quality of life should aim to improve methodological quality and enrich this area of study to confirm the effects of exercise on quality of life.

There are strengths and limitations to this study. On the one hand, the data extracted from eligible studies exhibited high quality, reflecting our rigorous efforts to collect relevant RCTs and the thoroughness with which our team ensured accuracy and consistency during data extraction. Moreover, the application of the GRADE approach provided a systematic and transparent assessment of the quality of the evidence, helping to identify the strengths and weaknesses of the included studies. However, some intervention results remain unclear or should be interpreted with caution. This is due to the low quality of evidence regarding depression and quality of sleep, as well as the limited number of available RCTs on exercise interventions for anxiety and sleep disorders in individuals with MCI. Furthermore, since the majority of studies did not include follow-up periods after the intervention, this analysis solely focused on the immediate effects of the exercise interventions, omitting the evaluation of the long-term maintenance effects. Lastly, considering that the retrieved experimental studies on this topic did not report on a broader range of neuropsychiatric symptoms, we focused only on depression, anxiety, and sleep disorders to illustrate the effect of exercise interventions on NPS in MCI patients. We anticipate that future experimental studies will pay greater attention to other NPS as well, thereby enabling a more comprehensive evaluation of the intervention effects.

## 5 Conclusion

Exercise has a small positive effect on depression in individuals with MCI. However, this is based on evidence of low quality. Among the various forms of exercise, mind–body exercises appear to be more effective in alleviating depression. While exercise may not significantly improve sleep quality in MCI patients, the supporting evidence is of very low quality. The quality of research in this area needs to be improved in order to further clarify the effects of the interventions. Additionally, inconsistencies in the structure of measurement tools prevented the conduct of a meta-analysis of the intervention effects. Therefore, more high-quality RCTs are required to validate the beneficial effects of exercise on anxiety and the quality of life of individuals with MCI.

## Data Availability

The original contributions presented in the study are included in the article/Supplementary material, further inquiries can be directed to the corresponding author.

## Footnotes

^1^gdt.gradepro.org
