# Supplementary material for: Exercise effects on neuropsychiatric symptoms and quality of life in mild cognitive impairment: a systematic review and meta-analysis
Source: Front Neurol. 2024 Oct 14;15:1447734. doi: 10.3389/fneur.2024.1447734 (PMC11513880; doi:10.3389/fneur.2024.1447734)
Supplement: Supplementary file 1 [file Table_1.DOCX]

# **Supplementary Table1** Search Strategy

| **Database** | **Searchs** | **Results** |
| --- | --- | --- |
| **Pubmed** | (((randomized controlled trial[Publication Type] OR randomized[Title/Abstract] OR placebo[Title/Abstract])) AND (("Cognitive Dysfunction"[Mesh]) OR (((((((((((((((((((((((((Cognitive Dysfunctions[Title/Abstract]) OR (Dysfunction, Cognitive[Title/Abstract])) OR (Dysfunctions, Cognitive[Title/Abstract])) OR (Cognitive Impairments[Title/Abstract])) OR (Cognitive Impairment[Title/Abstract])) OR (Impairment, Cognitive[Title/Abstract])) OR (Impairments, Cognitive[Title/Abstract])) OR (Cognitive Disorder[Title/Abstract])) OR (Cognitive Disorders[Title/Abstract])) OR (Disorder, Cognitive[Title/Abstract])) OR (Disorders, Cognitive[Title/Abstract])) OR (Mild Cognitive Impairment[Title/Abstract])) OR (Cognitive Impairment, Mild[Title/Abstract])) OR (Cognitive Impairments, Mild[Title/Abstract])) OR (Impairment, Mild Cognitive[Title/Abstract])) OR (Impairments, Mild Cognitive[Title/Abstract])) OR (Mild Cognitive Impairments[Title/Abstract])) OR (Cognitive Decline[Title/Abstract])) OR (Cognitive Declines[Title/Abstract])) OR (Decline, Cognitive[Title/Abstract])) OR (Declines, Cognitive[Title/Abstract])) OR (Mental Deterioration[Title/Abstract])) OR (Deterioration, Mental[Title/Abstract])) OR (Deteriorations, Mental[Title/Abstract])) OR (Mental Deteriorations[Title/Abstract])))) AND (("Exercise"[Mesh]) OR (((((((((((((((((((((((((((((((((((((((((((((((((((((((((((((((((((((((((((((((Football[Title/Abstract])) OR (Basketball[Title/Abstract])) OR (Tennis[Title/Abstract])) OR (Baseball[Title/Abstract])) OR (Volleyball[Title/Abstract])) OR (Badminton[Title/Abstract])) OR (Table Tennis[Title/Abstract])) OR (Swimming[Title/Abstract])) OR (Running[Title/Abstract])) OR (Cycling[Title/Abstract])) OR (Gymnastics[Title/Abstract])) OR (Ice Hockey[Title/Abstract])) OR (Figure Skating[Title/Abstract])) OR (Skiing[Title/Abstract])) OR (Snowboarding[Title/Abstract])) OR (Wrestling[Title/Abstract])) OR (Boxing[Title/Abstract])) OR (Martial Arts[Title/Abstract])) OR (Golf[Title/Abstract])) OR (Rugby[Title/Abstract])) OR (Cricket[Title/Abstract])) OR (Field Hockey[Title/Abstract])) OR (Sailing[Title/Abstract])) OR (Surfing[Title/Abstract])) OR (Rock Climbing[Title/Abstract])) OR (Archery[Title/Abstract])) OR (Equestrian[Title/Abstract])) OR (Fencing[Title/Abstract])) OR (Canoeing[Title/Abstract])) OR (Kayaking[Title/Abstract])) OR (Diving[Title/Abstract])) OR (Snorkeling[Title/Abstract])) OR (Water Polo[Title/Abstract])) OR (Triathlon[Title/Abstract])) OR (CrossFit[Title/Abstract])) OR (Judo[Title/Abstract])) OR (Taekwondo[Title/Abstract])) OR (Karate[Title/Abstract])) OR (Kung Fu[Title/Abstract])) OR (Hiking[Title/Abstract])) OR (Rock Climbing[Title/Abstract])) OR (Mountaineering[Title/Abstract])) OR (Skydiving[Title/Abstract])) OR (Paragliding[Title/Abstract])) OR (Horse Racing[Title/Abstract])) OR (Formula 1 Racing[Title/Abstract])) OR (Rally Racing[Title/Abstract])) OR (Synchronized Swimming[Title/Abstract])) OR (Cross-Country Skiing[Title/Abstract])) OR (Skateboarding[Title/Abstract])) OR (Roller Skating[Title/Abstract])) OR (Inline Skating[Title/Abstract])) OR (Parkour[Title/Abstract])) OR (Handball[Title/Abstract])) OR (Lacrosse[Title/Abstract])) OR (Polo[Title/Abstract])) OR (BMX[Title/Abstract])) OR (Archery Tag[Title/Abstract])) OR (Bocce Ball[Title/Abstract])) OR (Chess Boxing[Title/Abstract])) OR (Kabaddi[Title/Abstract])) OR (Sepak Takraw[Title/Abstract])) OR (Underwater Hockey[Title/Abstract])) OR (Floorball[Title/Abstract])) OR (Handball[Title/Abstract])) OR (Korfball[Title/Abstract])) OR (Paddleboarding[Title/Abstract])) OR (Powerlifting[Title/Abstract])) OR (Weightlifting[Title/Abstract])) OR (Sumo Wrestling[Title/Abstract])) OR (Cross Country Running[Title/Abstract])) OR (Canyoning[Title/Abstract])) OR (Freediving[Title/Abstract])) OR (Slacklining[Title/Abstract])) OR (Hang Gliding[Title/Abstract])) OR (Ice Skating[Title/Abstract])) OR (Rhythmic Gymnastics[Title/Abstract])) OR (Squash[Title/Abstract])) OR (Orienteering[Title/Abstract])) OR (Dragon Boat Racing[Title/Abstract])) OR (Jiu-Jitsu[Title/Abstract])) OR (Cross-country Mountain Biking[Title/Abstract])) OR (Water Skiing[Title/Abstract])) OR (Freestyle Skiing[Title/Abstract])) OR (Stretching[Title/Abstract])) OR (Aerobics[Title/Abstract])) OR (Yoga[Title/Abstract])) OR (Tai Chi[Title/Abstract])) OR (Eight Brocade[Title/Abstract])) OR (Jump rope[Title/Abstract])) OR (Resistance training[Title/Abstract])) OR (dance[Title/Abstract]))) | **1322** |
| **Database** | **Searchs** | **Results** |
| **Web of science** | TS=(Cognitive Dysfunction OR Cognitive Dysfunctions OR Dysfunction, Cognitive OR Dysfunctions, Cognitive OR Cognitive Impairments OR Cognitive Impairment OR Impairment, Cognitive OR Impairments, Cognitive OR Cognitive Disorder OR Cognitive Disorders OR Disorder, Cognitive OR Disorders, Cognitive OR Mild Cognitive Impairment OR Cognitive Impairment, Mild OR Cognitive Impairments, Mild OR Impairment, Mild Cognitive OR Impairments, Mild Cognitive OR Mild Cognitive Impairments OR Cognitive Decline OR Cognitive Declines OR Decline, Cognitive OR Declines, Cognitive OR Mental Deterioration OR Deterioration, Mental OR Deteriorations, Mental OR Mental Deteriorations) AND TS=(randomized controlled trial OR randomized OR placebo OR RCT) AND TS=(Exercise OR Exercises OR Physical Activity OR Activities, Physical OR Activity, Physical OR Physical Activities OR Exercise, Physical OR Exercises, Physical OR Physical Exercise OR Physical Exercises OR Acute Exercise OR Acute Exercises OR Exercise, Acute OR Exercises, Acute OR Exercise, Isometric OR Exercises, Isometric OR Isometric Exercises OR Isometric Exercise OR Exercise, Aerobic OR Aerobic Exercise OR Aerobic Exercises OR Exercises, Aerobic OR Exercise Training OR Exercise Trainings OR Training, Exercise OR Trainings, Exercise OR walking OR Track and Field OR Soccer OR Football OR Basketball OR Tennis OR Baseball OR Volleyball OR Badminton OR Table Tennis OR Swimming OR Running OR Cycling OR Gymnastics OR Ice Hockey OR Figure Skating OR Skiing OR Snowboarding OR Wrestling OR Boxing OR Martial Arts OR Golf OR Rugby OR Cricket OR Field Hockey OR Sailing OR Surfing OR Rock Climbing OR Archery OR Equestrian OR Fencing OR Canoeing OR Kayaking OR Diving OR Snorkeling OR Water Polo OR Triathlon OR CrossFit OR Judo OR Taekwondo OR Karate OR Kung Fu OR Hiking OR Rock Climbing OR Mountaineering OR Skydiving OR Paragliding OR Horse Racing OR Formula 1 Racing OR Rally Racing OR Synchronized Swimming OR Cross-Country Skiing OR Skateboarding OR Roller Skating OR Inline Skating OR Parkour OR Handball OR Lacrosse OR Polo OR BMX OR Archery Tag OR Bocce Ball OR Chess Boxing OR Kabaddi OR Sepak Takraw OR Underwater Hockey OR Floorball OR Handball OR Korfball OR Paddleboarding OR Powerlifting OR Weightlifting OR Sumo Wrestling OR Cross Country Running OR Canyoning OR Freediving OR Slacklining OR Hang Gliding OR Ice Skating OR Rhythmic Gymnastics OR Squash OR Orienteering OR Dragon Boat Racing OR Jiu-Jitsu OR Cross-country Mountain Biking OR Water Skiing OR Freestyle Skiing OR Stretching OR Aerobics OR Yoga OR Tai Chi OR Eight Brocade OR Jump rope OR Resistance training OR dance) | **5154** |
| **Embase** | ('Exercises':ab,ti OR 'Physical Activity':ab,ti OR 'Activities, Physical':ab,ti OR 'Activity, Physical':ab,ti OR 'Physical Activities':ab,ti OR 'Exercise, Physical':ab,ti OR 'Exercises, Physical':ab,ti OR 'Physical Exercise':ab,ti OR 'Physical Exercises':ab,ti OR 'Acute Exercise':ab,ti OR 'Acute Exercises':ab,ti OR 'Exercise, Acute':ab,ti OR 'Exercises, Acute':ab,ti OR 'Exercise, Isometric':ab,ti OR 'Exercises, Isometric':ab,ti OR 'Isometric Exercises':ab,ti OR 'Isometric Exercise':ab,ti OR 'Exercise, Aerobic':ab,ti OR 'Aerobic Exercise':ab,ti OR 'Aerobic Exercises':ab,ti OR 'Exercises, Aerobic':ab,ti OR 'Exercise Training':ab,ti OR 'Exercise Trainings':ab,ti OR 'Training, Exercise':ab,ti OR 'Trainings, Exercise':ab,ti OR 'walking':ab,ti OR 'Track and Field':ab,ti OR 'Soccer':ab,ti OR 'Football':ab,ti OR 'Basketball':ab,ti OR 'Tennis':ab,ti OR 'Baseball':ab,ti OR 'Volleyball':ab,ti OR 'Badminton':ab,ti OR 'Table Tennis':ab,ti OR 'Swimming':ab,ti OR 'Running':ab,ti OR 'Cycling':ab,ti OR 'Gymnastics':ab,ti OR 'Ice Hockey':ab,ti OR 'Figure Skating':ab,ti OR 'Skiing':ab,ti OR 'Snowboarding':ab,ti OR 'Wrestling':ab,ti OR 'Boxing':ab,ti OR 'Martial Arts':ab,ti OR 'Golf':ab,ti OR 'Rugby':ab,ti OR 'Cricket':ab,ti OR 'Field Hockey':ab,ti OR 'Sailing':ab,ti OR 'Surfing':ab,ti OR 'Rock Climbing':ab,ti OR 'Archery':ab,ti OR 'Equestrian':ab,ti OR 'Fencing':ab,ti OR 'Canoeing':ab,ti OR 'Kayaking':ab,ti OR 'Diving':ab,ti OR 'Snorkeling':ab,ti OR 'Water Polo':ab,ti OR 'Triathlon':ab,ti OR 'CrossFit':ab,ti OR 'Judo':ab,ti OR 'Taekwondo':ab,ti OR 'Karate':ab,ti OR 'Kung Fu':ab,ti OR 'Hiking':ab,ti OR 'Rock Climbing':ab,ti OR 'Mountaineering':ab,ti OR 'Skydiving':ab,ti OR 'Paragliding':ab,ti OR 'Horse Racing':ab,ti OR 'Formula 1 Racing':ab,ti OR 'Rally Racing':ab,ti OR 'Synchronized Swimming':ab,ti OR 'Cross-Country Skiing':ab,ti OR 'Skateboarding':ab,ti OR 'Roller Skating':ab,ti OR 'Inline Skating':ab,ti OR 'Parkour':ab,ti OR 'Handball':ab,ti OR 'Lacrosse':ab,ti OR 'Polo':ab,ti OR 'BMX':ab,ti OR 'Archery Tag':ab,ti OR 'Bocce Ball':ab,ti OR 'Chess Boxing':ab,ti OR 'Kabaddi':ab,ti OR | **1938** |
| **Database** | **Searchs** | **Results** |
| **Embase** | 'Sepak Takraw':ab,ti OR 'Underwater Hockey':ab,ti OR 'Floorball':ab,ti OR 'Handball':ab,ti OR 'Korfball':ab,ti OR 'Paddleboarding':ab,ti OR 'Powerlifting':ab,ti OR 'Weightlifting':ab,ti OR 'Sumo Wrestling':ab,ti OR 'Cross Country Running':ab,ti OR 'Canyoning':ab,ti OR 'Freediving':ab,ti OR 'Slacklining':ab,ti OR 'Hang Gliding':ab,ti OR 'Ice Skating':ab,ti OR 'Rhythmic Gymnastics':ab,ti OR 'Squash':ab,ti OR 'Orienteering':ab,ti OR 'Dragon Boat Racing':ab,ti OR 'Jiu-Jitsu':ab,ti OR 'Cross-country Mountain Biking':ab,ti OR 'Water Skiing':ab,ti OR 'Freestyle Skiing':ab,ti OR 'Stretching':ab,ti OR 'Aerobics':ab,ti OR 'Yoga':ab,ti OR 'Tai Chi':ab,ti OR 'Eight Brocade':ab,ti OR 'Jump rope':ab,ti OR 'Resistance training':ab,ti OR 'dance':ab,ti) AND ('Cognitive Dysfunctions':ab,ti OR 'Dysfunction, Cognitive ':ab,ti OR 'Dysfunctions, Cognitive':ab,ti OR 'Cognitive Impairments':ab,ti OR 'Cognitive Impairment':ab,ti OR 'Impairment, Cognitive':ab,ti OR 'Impairments, Cognitive':ab,ti OR 'Cognitive Disorder':ab,ti OR 'Cognitive Disorders':ab,ti OR 'Disorder, Cognitive':ab,ti OR 'Disorders, Cognitive':ab,ti OR 'Mild Cognitive Impairment':ab,ti OR 'Cognitive Impairment, Mild':ab,ti OR 'Cognitive Impairments, Mild':ab,ti OR 'Impairment, Mild Cognitive':ab,ti OR 'Impairments, Mild Cognitive':ab,ti OR 'Mild Cognitive Impairments':ab,ti OR 'Cognitive Decline':ab,ti OR 'Cognitive Declines':ab,ti OR 'Decline, Cognitive':ab,ti OR 'Declines, Cognitive':ab,ti OR 'Mental Deterioration':ab,ti OR 'Deterioration, Mental':ab,ti OR 'Deteriorations, Mental':ab,ti OR 'Mental Deteriorations':ab,ti) AND ('randomized controlled trial':ab,ti OR 'randomized':ab,ti OR 'placebo':ab,ti OR 'RCT':ab,ti) | **1938** |
| **Cocharane Library** | ((randomized controlled trial):ab,ti,kw OR (randomized):ab,ti,kw OR (placebo):ab,ti,kw OR (RCT) ) AND ((Cognitive Dysfunctions):ab,ti,kw OR (Dysfunction, Cognitive):ab,ti,kw OR (Dysfunctions, Cognitive):ab,ti,kw OR (Cognitive Impairments):ab,ti,kw OR (Cognitive Impairment):ab,ti,kw OR (Impairment, Cognitive):ab,ti,kw OR (Impairments, Cognitive):ab,ti,kw OR (Cognitive Disorder):ab,ti,kw OR (Cognitive Disorders):ab,ti,kw OR (Disorder, Cognitive):ab,ti,kw OR (Disorders, Cognitive):ab,ti,kw OR (Mild Cognitive Impairment):ab,ti,kw OR (Cognitive Impairment, Mild):ab,ti,kw OR (Cognitive Impairments, Mild):ab,ti,kw OR (Impairment, Mild Cognitive):ab,ti,kw OR (Impairments, Mild Cognitive):ab,ti,kw OR (Mild Cognitive Impairments):ab,ti,kw OR (Cognitive Decline):ab,ti,kw OR (Cognitive Declines):ab,ti,kw OR (Decline, Cognitive):ab,ti,kw OR (Declines, Cognitive):ab,ti,kw OR (Mental Deterioration):ab,ti,kw OR (Deterioration, Mental):ab,ti,kw OR (Deteriorations, Mental):ab,ti,kw OR (Mental Deteriorations)) AND ((Exercises):ab,ti,kw OR (Physical Activity):ab,ti,kw OR (Activities, Physical):ab,ti,kw OR (Activity, Physical):ab,ti,kw OR (Physical Activities):ab,ti,kw OR (Exercise, Physical):ab,ti,kw OR (Exercises, Physical):ab,ti,kw OR (Physical Exercise):ab,ti,kw OR (Physical Exercises):ab,ti,kw OR (Acute Exercise):ab,ti,kw OR (Acute Exercises):ab,ti,kw OR (Exercise, Acute):ab,ti,kw OR (Exercises, Acute):ab,ti,kw OR (Exercise, Isometric):ab,ti,kw OR (Exercises, Isometric):ab,ti,kw OR (Isometric Exercises):ab,ti,kw OR (Isometric Exercise):ab,ti,kw OR (Exercise, Aerobic):ab,ti,kw OR (Aerobic Exercise):ab,ti,kw OR (Aerobic Exercises):ab,ti,kw OR (Exercises, Aerobic):ab,ti,kw OR (Exercise Training):ab,ti,kw OR (Exercise Trainings):ab,ti,kw OR (Training, Exercise):ab,ti,kw OR (Trainings, Exercise):ab,ti,kw OR (walking):ab,ti,kw OR (Track and Field):ab,ti,kw OR (Soccer):ab,ti,kw OR (Football):ab,ti,kw OR (Basketball):ab,ti,kw OR (Tennis):ab,ti,kw OR (Baseball):ab,ti,kw OR (Volleyball):ab,ti,kw OR (Badminton):ab,ti,kw OR (Table Tennis):ab,ti,kw OR (Swimming):ab,ti,kw OR (Running):ab,ti,kw OR (Cycling):ab,ti,kw OR (Gymnastics):ab,ti,kw OR (Ice Hockey):ab,ti,kw OR (Figure Skating):ab,ti,kw OR (Skiing):ab,ti,kw OR (Snowboarding):ab,ti,kw OR (Wrestling):ab,ti,kw OR (Boxing):ab,ti,kw OR (Martial Arts):ab,ti,kw OR (Golf):ab,ti,kw OR (Rugby):ab,ti,kw OR (Cricket):ab,ti,kw OR (Field Hockey):ab,ti,kw OR (Sailing):ab,ti,kw OR (Surfing):ab,ti,kw OR (Rock Climbing):ab,ti,kw OR (Archery):ab,ti,kw OR (Equestrian):ab,ti,kw OR (Fencing):ab,ti,kw OR (Canoeing):ab,ti,kw OR (Kayaking):ab,ti,kw OR (Diving):ab,ti,kw OR (Snorkeling):ab,ti,kw OR (Water Polo):ab,ti,kw OR (Triathlon):ab,ti,kw OR (CrossFit):ab,ti,kw OR (Judo):ab,ti,kw OR (Taekwondo):ab,ti,kw OR (Karate):ab,ti,kw OR (Kung Fu):ab,ti,kw OR (Hiking):ab,ti,kw OR (Rock Climbing):ab,ti,kw OR (Mountaineering):ab,ti,kw OR (Skydiving):ab,ti,kw OR (Paragliding):ab,ti,kw OR (Horse Racing):ab,ti,kw OR (Formula 1 Racing):ab,ti,kw OR (Rally Racing):ab,ti,kw OR (Synchronized Swimming):ab,ti,kw OR (Cross-Country | **5498** |
| **Database** | **Searchs** | **Results** |
| **Cocharane Library** | Skiing):ab,ti,kw OR (Skateboarding):ab,ti,kw OR (Roller Skating):ab,ti,kw OR (Inline Skating):ab,ti,kw OR (Parkour):ab,ti,kw OR (Handball):ab,ti,kw OR (Lacrosse):ab,ti,kw OR (Polo):ab,ti,kw OR (BMX):ab,ti,kw OR (Archery Tag):ab,ti,kw OR (Bocce Ball):ab,ti,kw OR (Chess Boxing):ab,ti,kw OR (Kabaddi):ab,ti,kw OR (Sepak Takraw):ab,ti,kw OR (Underwater Hockey):ab,ti,kw OR (Floorball):ab,ti,kw OR (Handball):ab,ti,kw OR (Korfball):ab,ti,kw OR (Paddleboarding):ab,ti,kw OR (Powerlifting):ab,ti,kw OR (Weightlifting):ab,ti,kw OR (Sumo Wrestling):ab,ti,kw OR (Cross Country Running):ab,ti,kw OR (Canyoning):ab,ti,kw OR (Freediving):ab,ti,kw OR (Slacklining):ab,ti,kw OR (Hang Gliding):ab,ti,kw OR (Ice Skating):ab,ti,kw OR (Rhythmic Gymnastics):ab,ti,kw OR (Squash):ab,ti,kw OR (Orienteering):ab,ti,kw OR (Dragon Boat Racing):ab,ti,kw OR (Jiu-Jitsu):ab,ti,kw OR (Cross-country Mountain Biking):ab,ti,kw OR (Water Skiing):ab,ti,kw OR (Freestyle Skiing)) | **5498** |
| **Scopus** | ( TITLE-ABS-KEY ( cognitive AND dysfunction ) OR TITLE-ABS-KEY ( cognitive AND dysfunctions ) OR TITLE-ABS-KEY ( dysfunction, AND cognitive ) OR TITLE-ABS-KEY ( dysfunctions, AND cognitive ) OR TITLE-ABS-KEY ( cognitive AND impairments ) OR TITLE-ABS-KEY ( cognitive AND impairment ) OR TITLE-ABS-KEY ( impairment, AND cognitive ) OR TITLE-ABS-KEY ( impairments, AND cognitive ) OR TITLE-ABS-KEY ( cognitive AND disorder ) OR TITLE-ABS-KEY ( cognitive AND disorders ) OR TITLE-ABS-KEY ( disorder, AND cognitive ) OR TITLE-ABS-KEY ( disorders, AND cognitive ) OR TITLE-ABS-KEY ( mild AND cognitive AND impairment ) OR ( TITLE-ABS-KEY ( exercise ) OR TITLE-ABS-KEY ( exercises ) OR TITLE-ABS-KEY ( physical AND activity ) OR TITLE-ABS-KEY ( activities, AND physical ) OR TITLE-ABS-KEY ( activity, AND physical ) OR TITLE-ABS-KEY ( physical AND activities ) OR TITLE-ABS-KEY ( exercise, AND physical ) OR TITLE-ABS-KEY ( exercises, AND physical ) OR TITLE-ABS-KEY ( physical AND exercise ) OR TITLE-ABS-KEY ( physical AND exercises ) OR TITLE-ABS-KEY ( acute AND exercise ) OR TITLE-ABS-KEY ( acute AND exercises ) OR TITLE-ABS-KEY ( exercise, AND acute ) OR TITLE-ABS-KEY ( exercises, AND acute ) OR TITLE-ABS-KEY ( exercise, AND isometric ) OR TITLE-ABS-KEY ( exercises, AND isometric ) OR TITLE-ABS-KEY ( isometric AND exercises ) OR TITLE-ABS-KEY ( isometric AND exercise ) OR TITLE-ABS-KEY ( exercise, AND aerobic ) OR TITLE-ABS-KEY ( aerobic AND exercise ) OR TITLE-ABS-KEY ( aerobic AND exercises ) OR TITLE-ABS-KEY ( exercises, AND aerobic ) OR TITLE-ABS-KEY ( exercise AND training ) OR TITLE-ABS-KEY ( exercise AND trainings ) OR TITLE-ABS-KEY ( training, AND exercise ) OR TITLE-ABS-KEY ( trainings, AND exercise ) OR TITLE-ABS-KEY ( walking ) OR TITLE-ABS-KEY ( track AND field ) OR TITLE-ABS-KEY ( soccer ) OR TITLE-ABS-KEY ( football ) OR TITLE-ABS-KEY ( basketball ) OR TITLE-ABS-KEY ( tennis ) OR TITLE-ABS-KEY ( baseball ) OR TITLE-ABS-KEY ( volleyball ) OR TITLE-ABS-KEY ( badminton ) OR TITLE-ABS-KEY ( table AND tennis ) OR TITLE-ABS-KEY ( swimming ) OR TITLE-ABS-KEY ( running ) OR TITLE-ABS-KEY ( cycling ) OR TITLE-ABS-KEY ( gymnastics ) OR TITLE-ABS-KEY ( ice AND hockey ) OR TITLE-ABS-KEY ( figure AND skating ) OR TITLE-ABS-KEY ( skiing ) OR TITLE-ABS-KEY ( snowboarding ) OR TITLE-ABS-KEY ( wrestling ) OR TITLE-ABS-KEY ( boxing ) OR TITLE-ABS-KEY ( martial AND arts ) OR TITLE-ABS-KEY ( golf ) OR TITLE-ABS-KEY ( rugby ) OR TITLE-ABS-KEY ( cricket ) OR TITLE-ABS-KEY ( field AND hockey ) OR TITLE-ABS-KEY ( sailing ) OR TITLE-ABS-KEY ( surfing ) OR TITLE-ABS-KEY ( rock AND climbing ) OR TITLE-ABS-KEY ( archery ) OR TITLE-ABS-KEY ( equestrian ) OR TITLE-ABS-KEY ( fencing ) OR TITLE-ABS-KEY ( canoeing ) OR TITLE-ABS-KEY ( kayaking ) OR TITLE-ABS-KEY ( diving ) OR TITLE-ABS-KEY ( snorkeling ) OR TITLE-ABS-KEY ( water AND polo ) OR TITLE-ABS-KEY ( triathlon ) OR TITLE-ABS-KEY ( crossfit ) OR TITLE-ABS-KEY ( judo ) OR TITLE-ABS-KEY ( taekwondo ) OR TITLE-ABS-KEY ( karate ) OR TITLE-ABS-KEY ( kung AND fu ) OR TITLE-ABS-KEY ( hiking ) OR TITLE-ABS-KEY ( rock AND climbing ) OR TITLE-ABS-KEY ( mountaineering ) OR TITLE-ABS-KEY ( skydiving ) OR TITLE-ABS-KEY ( paragliding ) OR TITLE-ABS-KEY ( horse AND racing ) OR TITLE-ABS-KEY | **6338** |
| **Database** | **Searchs** | **Results** |
| **Scopus** | ( formula 1 racing ) OR TITLE-ABS-KEY ( rally AND racing ) OR TITLE-ABS-KEY ( synchronized AND swimming ) OR TITLE-ABS-KEY ( cross-country AND skiing ) OR TITLE-ABS-KEY ( skateboarding ) OR TITLE-ABS-KEY ( roller AND skating ) OR TITLE-ABS-KEY ( inline AND skating ) OR TITLE-ABS-KEY ( parkour ) OR TITLE-ABS-KEY ( handball ) OR TITLE-ABS-KEY ( lacrosse ) OR TITLE-ABS-KEY ( polo ) OR TITLE-ABS-KEY ( bmx ) OR TITLE-ABS-KEY ( archery AND tag ) OR TITLE-ABS-KEY ( bocce AND ball ) OR TITLE-ABS-KEY ( chess AND boxing ) OR TITLE-ABS-KEY ( kabaddi ) OR TITLE-ABS-KEY ( sepak AND takraw ) OR TITLE-ABS-KEY ( underwater AND hockey ) OR TITLE-ABS-KEY ( floorball ) OR TITLE-ABS-KEY ( handball ) OR TITLE-ABS-KEY ( korfball ) OR TITLE-ABS-KEY ( paddleboarding ) OR TITLE-ABS-KEY ( powerlifting ) OR TITLE-ABS-KEY ( weightlifting ) OR TITLE-ABS-KEY ( sumo AND wrestling ) OR TITLE-ABS-KEY ( cross AND country AND running ) OR TITLE-ABS-KEY ( canyoning ) OR TITLE-ABS-KEY ( freediving ) OR TITLE-ABS-KEY ( slacklining ) OR TITLE-ABS-KEY ( hang AND gliding ) OR TITLE-ABS-KEY ( ice AND skating ) OR TITLE-ABS-KEY ( rhythmic AND gymnastics ) OR TITLE-ABS-KEY ( squash ) OR TITLE-ABS-KEY ( orienteering ) OR TITLE-ABS-KEY ( dragon AND boat AND racing ) OR TITLE-ABS-KEY ( jiu-jitsu ) OR TITLE-ABS-KEY ( cross-country AND mountain AND biking ) OR TITLE-ABS-KEY ( water AND skiing ) OR TITLE-ABS-KEY ( freestyle AND skiing ) OR TITLE-ABS-KEY ( stretching ) OR TITLE-ABS-KEY ( aerobics ) OR TITLE-ABS-KEY ( yoga ) OR TITLE-ABS-KEY ( tai AND chi ) OR TITLE-ABS-KEY ( eight AND brocade ) OR TITLE-ABS-KEY ( jump AND rope ) OR TITLE-ABS-KEY ( resistance AND training ) OR TITLE-ABS-KEY ( dance ) ) AND ( TITLE-ABS-KEY ( cognitive AND dysfunction ) OR TITLE-ABS-KEY ( cognitive AND dysfunctions ) OR TITLE-ABS-KEY ( dysfunction, AND cognitive ) OR TITLE-ABS-KEY ( dysfunctions, AND cognitive ) OR TITLE-ABS-KEY ( cognitive AND impairments ) OR TITLE-ABS-KEY ( cognitive AND impairment ) OR TITLE-ABS-KEY ( impairment, AND cognitive ) OR TITLE-ABS-KEY ( impairments, AND cognitive ) OR TITLE-ABS-KEY ( cognitive AND disorder ) OR TITLE-ABS-KEY ( cognitive AND disorders ) OR TITLE-ABS-KEY ( disorder, AND cognitive ) OR TITLE-ABS-KEY ( disorders, AND cognitive ) OR TITLE-ABS-KEY ( mild AND cognitive AND impairment ) OR TITLE-ABS-KEY ( cognitive AND impairment, AND mild ) OR TITLE-ABS-KEY ( cognitive AND impairments, AND mild ) OR TITLE-ABS-KEY ( impairment, AND mild AND cognitive ) OR TITLE-ABS-KEY ( impairments, AND mild AND cognitive ) OR TITLE-ABS-KEY ( mild AND cognitive AND impairments ) OR TITLE-ABS-KEY ( cognitive AND declines ) OR TITLE-ABS-KEY ( decline, AND cognitive ) OR TITLE-ABS-KEY ( declines, AND cognitive ) OR TITLE-ABS-KEY ( mental AND deterioration ) OR TITLE-ABS-KEY ( deterioration, AND mental ) OR TITLE-ABS-KEY ( deteriorations, AND mental ) OR TITLE-ABS-KEY ( mental AND deteriorations ) ) ]and ( TITLE-ABS-KEY ( randomized AND controlled AND trial ) OR TITLE-ABS-KEY ( randomized ) OR TITLE-ABS-KEY ( placebo ) OR TITLE-ABS-KEY ( rct ) ) | **6338** |
| **Medline** | AB ( Cognitive Dysfunction OR Cognitive Dysfunctions OR Dysfunction, Cognitive OR Dysfunctions, Cognitive OR Cognitive Impairments OR Cognitive Impairment OR Impairment, Cognitive OR Impairments, Cognitive OR Cognitive Disorder OR Cognitive Disorders OR Disorder, Cognitive OR Disorders, Cognitive OR Mild Cognitive Impairment OR Cognitive Impairment, Mild OR Cognitive Impairments, Mild OR Impairment, Mild Cognitive OR Impairments, Mild Cognitive OR Mild Cognitive Impairments OR Cognitive Decline OR Cognitive Declines OR Decline, Cognitive OR Declines, Cognitive OR Mental Deterioration OR Deterioration, Mental OR Deteriorations, Mental OR Mental Deteriorations ) AND AB ( Exercise OR Exercises OR Physical Activity OR Activities, Physical OR Activity, Physical OR Physical Activities OR Exercise, Physical OR Exercises, Physical OR Physical Exercise OR Physical Exercises OR Acute Exercise OR Acute Exercises OR Exercise, Acute OR Exercises, Acute OR Exercise, Isometric OR Exercises, Isometric OR Isometric Exercises OR Isometric Exercise OR Exercise, Aerobic OR Aerobic Exercise OR Aerobic Exercises OR Exercises, Aerobic OR Exercise Training OR Exercise Trainings OR Training, Exercise OR Trainings, Exercise ) AND AB ( randomized controlled trial OR randomized OR placebo OR RCT ) | **730** |
| **Database** | **Searchs** | **Results** |
| **APA PsycINFO** | AB ( randomized controlled trial OR randomized OR placebo OR RCT ) AND AB ( Exercise OR Exercises OR walking OR Track and Field OR Soccer OR Football OR Basketball OR Tennis OR Baseball OR Volleyball OR Badminton OR Table Tennis OR Swimming OR Running OR Cycling OR Gymnastics OR Ice Hockey OR Figure Skating OR Skiing OR Snowboarding OR Wrestling OR Boxing OR Golf OR Rugby OR Cricket OR Field Hockey OR Sailing OR Surfing OR Rock Climbing OR Archery OR Equestrian OR Fencing OR Canoeing OR Kayaking OR Diving OR Triathlon OR CrossFit OR Judo OR Taekwondo OR Karate OR Kung Fu OR Hiking OR Rock Climbing OR Mountaineering OR Skydiving OR Paragliding OR Horse Racing OR Synchronized Swimming OR Floorball OR Handball OR Korfball OR Paddleboarding OR Powerlifting OR Weightlifting OR Sumo Wrestling OR Cross Country Running OR Canyoning OR Freediving OR Slacklining OR Hang Gliding OR Ice Skating OR Rhythmic Gymnastics OR Squash OR Orienteering OR Dragon Boat Racing OR Cross-country Mountain Biking OR Water Skiing OR Freestyle Skiing OR Stretching OR Aerobics OR Yoga OR Tai Chi OR Eight Brocade OR Jump rope OR Resistance training OR dance ) AND AB ( Cognitive Dysfunction OR Cognitive Dysfunctions OR Dysfunction, Cognitive OR Dysfunctions, Cognitive OR Cognitive Impairments OR Cognitive Impairment OR Impairment, Cognitive OR Impairments, Cognitive OR Cognitive Disorder OR Cognitive Disorders OR Disorder, Cognitive OR Disorders, Cognitive OR Mild Cognitive Impairment OR Cognitive Impairment, Mild OR Cognitive Impairments, Mild OR Impairment, Mild Cognitive OR Impairments, Mild Cognitive OR Mild Cognitive Impairments OR Cognitive Decline OR Cognitive Declines OR Decline, Cognitive OR Declines, Cognitive OR Mental Deterioration OR Deterioration, Mental OR Deteriorations) | **369** |
| **Internat-ional Clinical Trials Registry Platform** | TS=(Cognitive Dysfunction OR Cognitive Dysfunctions OR Dysfunction, Cognitive OR Dysfunctions, Cognitive OR Cognitive Impairments OR Cognitive Impairment OR Impairment, Cognitive OR Impairments, Cognitive OR Cognitive Disorder OR Cognitive Disorders OR Disorder, Cognitive OR Disorders, Cognitive OR Mild Cognitive Impairment OR Cognitive Impairment, Mild OR Cognitive Impairments, Mild OR Impairment, Mild Cognitive OR Impairments, Mild Cognitive OR Mild Cognitive Impairments OR Cognitive Decline OR Cognitive Declines OR Decline, Cognitive OR Declines, Cognitive OR Mental Deterioration OR Deterioration, Mental OR Deteriorations, Mental OR Mental Deteriorations) AND TS=(randomized controlled trial OR randomized OR placebo OR RCT) AND TS=(Exercise OR Exercises OR Physical Activity OR Activities, Physical OR Activity, Physical OR Physical Activities OR Exercise, Physical OR Exercises, Physical OR Physical Exercise OR Physical Exercises OR Acute Exercise OR Acute Exercises OR Exercise, Acute OR Exercises, Acute OR Exercise, Isometric OR Exercises, Isometric OR Isometric Exercises OR Isometric Exercise OR Exercise, Aerobic OR Aerobic Exercise OR Aerobic Exercises OR Exercises, Aerobic OR Exercise Training OR Exercise Trainings OR Training, Exercise OR Trainings, Exercise OR walking OR Track and Field OR Soccer OR Football OR Basketball OR Tennis OR Baseball OR Volleyball OR Badminton OR Table Tennis OR Swimming OR Running OR Cycling OR Gymnastics OR Ice Hockey OR Figure Skating OR Skiing OR Snowboarding OR Wrestling OR Boxing OR Martial Arts OR Golf OR Rugby OR Cricket OR Field Hockey OR Sailing OR Surfing OR Rock Climbing OR Archery OR Equestrian OR Fencing OR Canoeing OR Kayaking OR Diving OR Snorkeling OR Water Polo OR Triathlon OR CrossFit OR Judo OR Taekwondo OR Karate OR Kung Fu OR Hiking OR Rock Climbing OR Mountaineering OR Skydiving OR Paragliding OR Horse Racing OR Formula 1 Racing OR Rally Racing OR Synchronized Swimming OR Cross-Country Skiing OR Skateboarding OR Roller Skating OR Inline Skating OR Parkour OR Handball OR Lacrosse OR Polo OR BMX OR Archery Tag OR Bocce Ball OR Chess Boxing OR Kabaddi OR Sepak Takraw OR Underwater Hockey OR Floorball OR Handball OR Korfball OR Paddleboarding OR Powerlifting OR Weightlifting 113OR Sumo Wrestling OR Cross Country Running OR Canyoning OR Freediving OR Slacklining OR Hang Gliding OR Ice Skating OR Rhythmic Gymnastics OR Squash OR Orienteering OR Dragon Boat Racing OR Jiu-Jitsu OR Cross-country Mountain Biking OR Water Skiing OR Freestyle Skiing OR Stretching OR Aerobics OR Yoga OR Tai Chi OR Eight Brocade OR Jump rope OR Resistance training OR dance) | **113** |
